# Copper Metabolism-Related Cell Death in Kidney Diseases: Molecular Mechanisms, Disease-Specific Evidence, and Translational Implications

**DOI:** 10.3390/ijms27167071

**Published:** 2026-08-07

**Authors:** Wei Shao, Qingguo Wang, Yanting Liu, Lingling Li, Xiaomin Li, Xueqian Wang, Fafeng Cheng

**Affiliations:** 1 School of Traditional Chinese Medicine, Beijing University of Chinese Medicine, Beijing 100029, China; 20250941019@bucm.edu.cn (W.S.);; 2 Institute of Basic Theory for Chinese Medicine, China Academy of Chinese Medical Sciences, Beijing 100700, China

**Keywords:** kidney diseases, copper dyshomeostasis, copper metabolism-related cell death, cuproptosis, protein lipoylation, acute kidney injury, renal fibrosis

## Abstract

Copper is essential for mitochondrial respiration, antioxidant defense, extracellular matrix maturation, and cellular signaling, yet disturbances in its abundance or intracellular distribution can damage the kidney through mechanistically distinct pathways. Cuproptosis is a specific copper-dependent form of regulated cell death in which copper binds lipoylated mitochondrial proteins, promotes aggregation of tricarboxylic acid cycle components, destabilizes iron–sulfur cluster proteins, and elicits FDX1- and protein lipoylation-dependent proteotoxic stress. This mechanism should be distinguished from broader copper-associated injury, including redox imbalance, glutathione depletion, respiratory-chain inhibition, senescence, apoptosis, and lysyl oxidase-mediated matrix remodeling. This narrative review examines how renal copper uptake, trafficking, and compartmentalization interact with cell-specific metabolism to shape copper-related cell fates across acute kidney injury, nephrotoxicity, renal ischemia–reperfusion injury, crystal- and lipid-related tubular injury, diabetic kidney disease, podocyte injury, chronic kidney disease and renal fibrosis, end-stage renal disease, renal cell carcinoma, and hereditary copper disorders. Mechanistic evidence is strongest in selected acute tubular, crystal-injury, and renal cancer models, in which transporter manipulation, DLAT oligomerization, iron–sulfur perturbation, or functional rescue has been demonstrated. In chronic kidney disease and fibrosis, copper-DLAT interactions, complex IV inhibition, COMMD1-SOD1 dysfunction, and ATP7A-FBLN4-LOX signaling establish pathogenic copper dependence but do not yet demonstrate a complete canonical cuproptosis pathway. By integrating disease-specific evidence with the molecular determinants of copper handling and protein lipoylation, this review identifies current therapeutic opportunities, candidate biomarkers, and key research priorities while preserving the distinction between cuproptosis and other forms of copper-associated kidney injury.

## 1. Introduction

The kidney is particularly sensitive to disturbances in metal homeostasis because it filters plasma, reabsorbs solutes, concentrates urine, and relies on mitochondria-rich epithelial cells to sustain energy-intensive transport. Copper is required for cytochrome c oxidase, superoxide dismutase 1 (SOD1), lysyl oxidase (LOX), and numerous redox and biosynthetic enzymes. However, copper excess, defective efflux, abnormal chaperoning, or redistribution among the cytosol, mitochondria, lysosomes, and Golgi apparatus can transform an essential micronutrient into a driver of renal injury. Consistent with this dual role, copper dyshomeostasis has been implicated in acute kidney injury (AKI), chronic kidney disease (CKD), diabetic kidney disease (DKD), renal fibrosis, nephrolithiasis, transplantation injury, and renal cancer [1,2,3,4].

The discovery of cuproptosis established a molecular framework for one specific form of copper-dependent cell death. In the canonical pathway, mitochondrial copper binds lipoylated tricarboxylic acid (TCA) cycle proteins, particularly dihydrolipoamide S-acetyltransferase (DLAT), promotes their abnormal aggregation, destabilizes iron–sulfur (Fe-S) cluster proteins, and induces proteotoxic stress that depends on FDX1 and the mitochondrial protein lipoylation pathway [5]. This mechanism is distinct from copper-mediated redox cycling, glutathione (GSH) depletion, complex IV inhibition, mitochondrial membrane depolarization, apoptosis, and ferroptosis, although these processes may coexist in injured renal cells [6,7].

The renal literature encompasses several mechanistically different copper-dependent lesions. Elevated tissue copper, altered expression of cuproptosis-related genes, oxidative stress, and mitochondrial dysfunction indicate copper exposure or susceptibility, but do not by themselves establish execution of cuproptosis. This distinction is especially important when evidence from AKI, cisplatin nephrotoxicity, ischemia–reperfusion injury, nonrenal fibrosis models, or renal cancer is considered in relation to chronic fibrotic CKD, where disease- and cell-specific validation remains limited [1,5,8,9,10,11].

**Figure 1:**
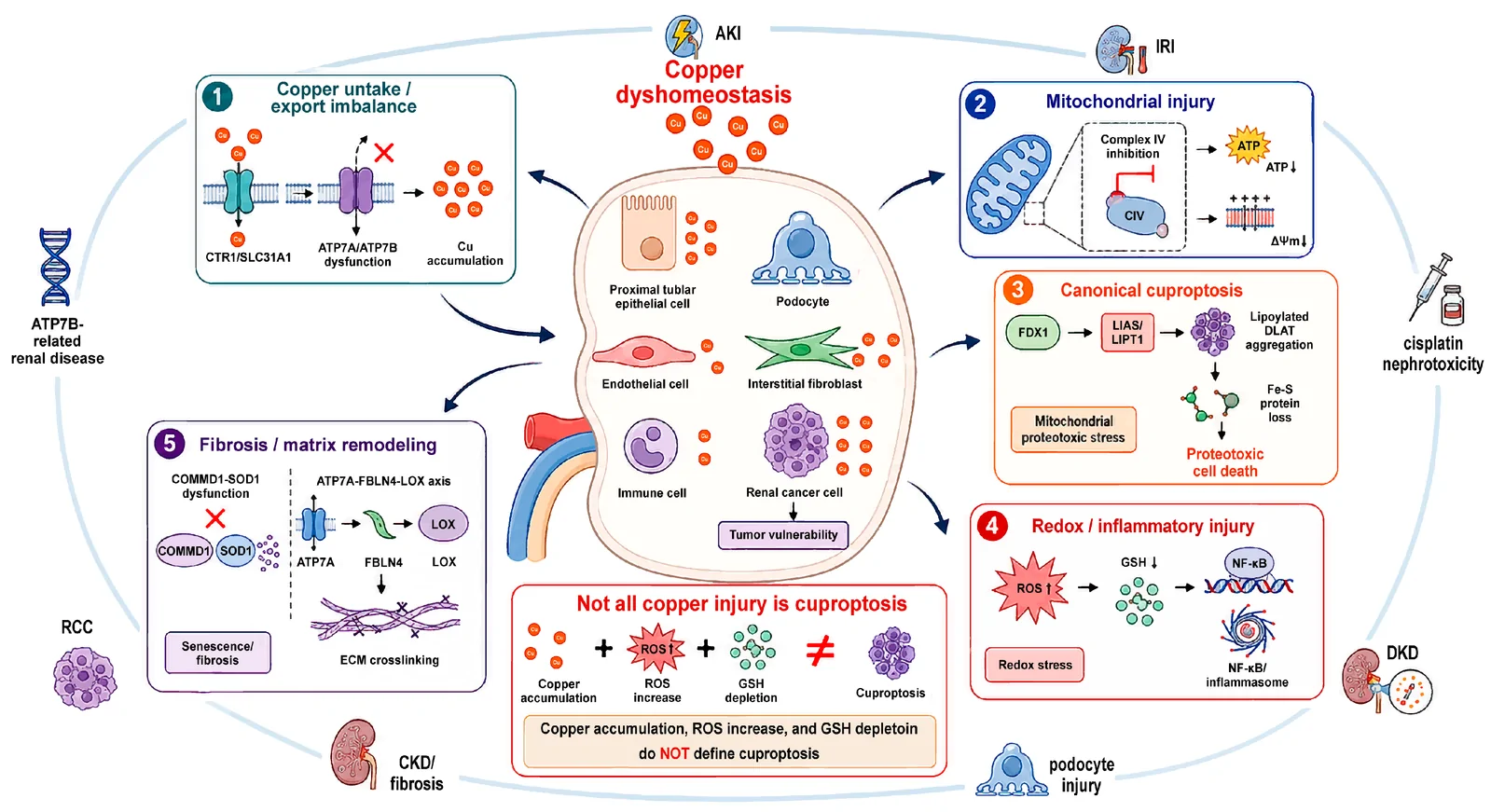
Conceptual framework of copper metabolism-related injury in kidney diseases. Renal copper dyshomeostasis may drive diverse injury programs, including mitochondrial dysfunction, oxidative stress, inflammatory activation, senescence, fibrosis and matrix remodeling, and tumor vulnerability. Canonical cuproptosis is depicted as a distinct and narrower mitochondrial proteotoxic pathway that requires FDX1- and protein lipoylation-dependent aggregation of lipoylated TCA-cycle proteins and destabilization of Fe-S proteins. Thus, copper accumulation, ROS elevation, or GSH depletion alone does not define cuproptosis.

In this review, “copper metabolism-related cell death” is used as an umbrella concept encompassing cell death and maladaptive cell fates caused by copper overload, deficiency, mislocalization, transporter dysfunction, or altered copper-dependent enzyme activity. The term “cuproptosis” is reserved for the FDX1- and protein lipoylation-dependent mitochondrial proteotoxic program [1,2,3,5]. The review first examines the principal pathophysiological interfaces between copper dyshomeostasis and kidney injury, then discusses renal copper handling and cell-specific susceptibility, evaluates disease-specific evidence, integrates shared and divergent mechanisms, and considers therapeutic and translational implications [12,13,14]. This scope and terminology are summarized in the conceptual framework shown in Figure 1.

**Review scope and literature search.** This critical narrative review was based on a PubMed/MEDLINE search from database inception through 20 July 2026. Search terms combined copper- and cuproptosis-related concepts (“cuproptosis”, “copper-dependent cell death”, “copper homeostasis”, “copper toxicity”, FDX1, LIAS, LIPT1, DLD, DLAT, SLC31A1/CTR1, ATP7A, ATP7B, iron–sulfur/Fe-S, and protein lipoylation) with kidney-related terms (“kidney”, “renal”, CKD, AKI, renal fibrosis, diabetic kidney disease, ischemia–reperfusion injury, cisplatin nephrotoxicity, nephrolithiasis, podocyte, and renal cell carcinoma). Reference lists of relevant primary studies and reviews were also examined. Kidney-specific original studies were prioritized; nonrenal studies were used mainly to define the canonical mechanism or to address areas in which renal evidence was unavailable. Bioinformatics studies were included as hypothesis-generating evidence rather than direct proof of cell death. Study selection emphasized mechanistic relevance, disease specificity, and depth of experimental validation rather than quantitative synthesis.

## 2. Pathophysiological Interfaces Between Copper Dyshomeostasis and Kidney Injury

### 2.1. Inflammation and Immune Remodeling

Inflammation amplifies both acute and chronic kidney injury and can be influenced by copper-dependent changes in redox state and cellular metabolism. Excess mitochondrial reactive oxygen species (mtROS) can activate NF-kappaB and inflammasome signaling; damaged mitochondria can release mitochondrial DNA and other danger-associated molecular patterns; and altered copper availability can reshape macrophage and lymphocyte metabolism [15,16,17,18,19]. These interactions are relevant to sepsis-associated AKI, ischemia–reperfusion injury, DKD, and renal cancer, although inflammation is not itself a defining feature of cuproptosis [5,20,21].

In sepsis-induced AKI, reduced GSH decreased renal copper content and SLC31A1 expression and was accompanied by lower oxidative stress, inflammatory cytokine production, and kidney dysfunction [17]. These findings support a copper-redox-inflammatory axis, but the measured endpoints did not establish the complete lipoylated-protein and Fe-S signature of cuproptosis. In DKD, transcriptomic studies have linked NLRP3, immune infiltration, and cuproptosis-related genes including LIAS, LIPT1, DLD, DBT, and DLST [22,23]. Such associations may aid molecular stratification, but they do not define a cell-death mechanism [15,24].

Renal cancer presents a different immunological context. Inducing cuproptosis in tumor cells may be therapeutically beneficial, whereas cuproptosis in antitumor immune cells may compromise host defense [18,19,25]. In renal cell carcinoma, iron-driven stabilization of FDX1 in CD8+ T cells promoted cuproptosis and impaired antitumor immunity, whereas restoration of ZDHHC12-dependent FDX1 palmitoylation rescued T-cell function and improved responses to PD-1 blockade [18]. The biological consequence of cuproptosis therefore depends on the affected cell population and therapeutic objective [26,27,28].

### 2.2. Oxidative Stress and Redox Imbalance

The kidney has a high oxygen demand and substantial mitochondrial content, making it particularly vulnerable to redox disruption. Copper can participate in redox cycling, consume thiol antioxidants, alter SOD1 activity, and disturb respiratory-chain function. Copper overload may therefore increase ROS, reduce GSH availability, damage mitochondrial macromolecules, and promote lipid peroxidation. These changes can engage several regulated cell-death programs, including apoptosis, pyroptosis, ferroptosis, necroptosis, and cuproptosis [15,29,30,31,32].

Oxidative stress is best viewed as a shared component of copper-associated injury rather than a specific marker of cuproptosis. In human CKD samples and experimental fibrotic kidneys, pathological copper overload impaired SOD1 homodimerization through a COMMD1-dependent mechanism. Lowering intracellular copper restored SOD1 activity, reduced ROS, and attenuated senescence and fibrosis [33]. This study defines a copper-dependent redox mechanism, but not the lipoylated-protein aggregation, Fe-S destabilization, and FDX1/protein lipoylation dependence that characterize canonical cuproptosis [34].

A similar distinction applies to GSH depletion and Fenton-like reactions. GSH buffers labile copper and supports GPX4-dependent protection from ferroptosis; its depletion can therefore increase copper availability, oxidative stress, and lipid peroxidation simultaneously [5,15,35]. Interventions that restore GSH may improve renal injury through several pathways, as shown in sepsis-associated AKI [17]. Their effects cannot be assigned specifically to cuproptosis unless the canonical mitochondrial proteotoxic changes are also reversed [36].

### 2.3. Renal Parenchymal and Endothelial Vulnerability

Renal tubular epithelial cells are prominent targets of copper-associated injury because they are rich in mitochondria, directly exposed to filtered and reabsorbed solutes, and highly dependent on oxidative phosphorylation [1,2,3]. Accordingly, most nonmalignant renal studies have used proximal tubular cell lines or tubular injury models. Increased SLC31A1-mediated uptake, impaired ATP7A/ATP7B-dependent export, and abnormal mitochondrial copper handling can reduce ATP production, depolarize mitochondrial membranes, and promote epithelial cell loss or maladaptive repair [8,37,38,39].

Podocytes are metabolically specialized epithelial cells that require mitochondrial energy to maintain the actin cytoskeleton and glomerular filtration barrier. Copper accumulation and adriamycin exposure have been associated with mitochondrial dysfunction, cytoskeletal disruption, and a cuproptosis-related injury phenotype; reducing copper accumulation and deleting CXCL5 attenuated podocyte damage [40]. The functional evidence is disease relevant, although the relationship to DLAT aggregation, Fe-S protein destabilization, and FDX1/protein lipoylation dependence remains incompletely defined [41].

Endothelial injury contributes to AKI, CKD, DKD, transplantation injury, and renal cancer, and multiple regulated cell-death pathways participate in diabetic and renal endothelial dysfunction [1,2,15,42]. Copper-dependent redox and mitochondrial disturbances are plausible contributors, but direct evidence for endothelial cuproptosis in kidney disease remains scarce. Most renal studies have focused on tubular epithelial cells, whole-kidney tissue, podocytes, or tumor cells [1,2,3]. Endothelial dysfunction should therefore be regarded as a potential consequence of copper dyshomeostasis rather than an established endothelial cuproptosis phenotype [43].

Together, inflammation, redox imbalance, and parenchymal or endothelial injury provide the principal pathophysiological interfaces through which copper dyshomeostasis may influence kidney disease [1,15]. Their biological significance depends on how copper is transported and compartmentalized, which mitochondrial substrates are available, and which renal cell population is affected. These determinants are considered in the next section [44].

## 3. Renal Copper Homeostasis and Determinants of Copper-Related Cell Fate

### 3.1. Renal Copper Uptake, Trafficking, and Compartmentalization

The biological effects of copper are determined not only by total renal copper content but also by copper speciation, intracellular availability, and subcellular distribution. Renal tubular epithelial cells require coordinated uptake, chaperoning, organelle delivery, and export to avoid both copper deficiency and copper overload. SLC31A1/CTR1 is the major high-affinity copper importer and has been implicated in tubular copper accumulation during cisplatin nephrotoxicity and ischemia–reperfusion injury. Increased SLC31A1 expression or stability enhanced intracellular copper accumulation, whereas SLC31A1 knockdown or lysosomal degradation reduced mitochondrial dysfunction and renal injury [8,37,45,46,47,48,49].

After cellular entry, copper is transferred by dedicated chaperones rather than remaining freely distributed in the cytosol. COX17 supports mitochondrial copper handling and cytochrome c oxidase activity. In experimental renal fibrosis, impaired COX17-dependent copper homeostasis increased mitochondrial copper accumulation, inhibited respiratory complex IV, and aggravated mitochondrial dysfunction, apoptosis, and fibrosis [50]. ATP7A and ATP7B regulate copper export and delivery to the secretory pathway. ATP7B suppression has been linked to tubular copper accumulation in lipid-related renal injury, whereas ATP7A-mediated copper delivery in the Golgi activates LOX and promotes extracellular matrix crosslinking during renal fibrosis [51,52,53].

Subcellular localization therefore helps determine the dominant lesion. Mitochondrial copper can disrupt respiratory-chain function, protein lipoylation, Fe-S protein stability, and proteostasis; Golgi copper can activate secretory cuproenzymes such as LOX; and cytosolic copper can alter antioxidant enzymes and redox-sensitive signaling. Pathological copper overload, for example, promoted COMMD1 binding to SOD1 and impaired SOD1 homodimerization, thereby increasing oxidative stress, senescence, and renal fibrosis [33]. These pathways arise from copper dyshomeostasis but should not be collapsed into a single mechanism [54,55].

**Figure 2:**
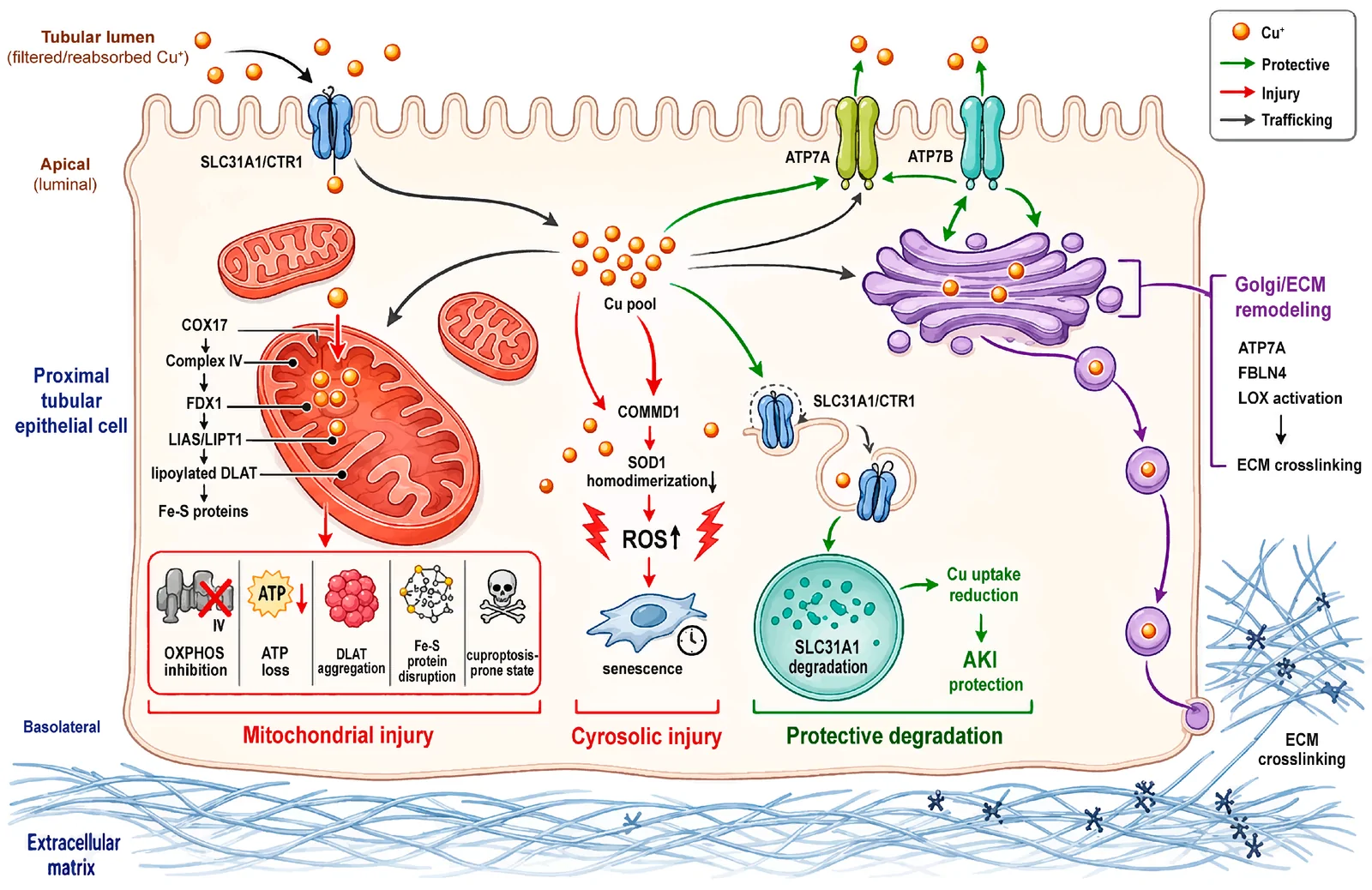
Renal copper handling and organelle-specific copper injury programs. Renal tubular epithelial cells regulate copper uptake, intracellular trafficking, mitochondrial delivery, Golgi transport, export, and degradation. The biological consequences of copper dyshomeostasis depend on its subcellular localization. Mitochondrial copper may disrupt respiratory-chain function, lipoylated DLAT assembly, and Fe-S protein stability; cytosolic copper may impair SOD1-dependent redox homeostasis and promote senescence; and Golgi-localized copper may enhance LOX-dependent extracellular matrix remodeling. In parallel, SLC31A1/CTR1 degradation may reduce copper uptake and confer protection in acute kidney injury.

Clinical and genetic observations reinforce the importance of renal copper handling. Marked tubular copper deposition has been reported in a patient with a heterozygous ATP7B variant, renal salt wasting, and chronic kidney dysfunction in the absence of a classic systemic Wilson disease phenotype [56]. Work on Wilson disease further emphasizes the relevance of copper speciation, interorgan flux, and barrier-mediated distribution to renal tubular injury [57]. Copper deposition or transporter dysfunction, however, does not by itself determine whether cells undergo adaptive dysfunction, oxidative injury, senescence, apoptosis, or cuproptosis [8,58,59]. These compartment-specific routes and injury programs are summarized in Figure 2.

### 3.2. Mitochondrial Protein Lipoylation and the Molecular Basis of Cuproptosis

Cuproptosis is one specific outcome of disturbed mitochondrial copper homeostasis. It preferentially affects cells with active mitochondrial respiration and abundant lipoylated TCA-cycle proteins. In the canonical pathway, copper binds lipoylated mitochondrial proteins, particularly DLAT, promotes their abnormal oligomerization or aggregation, destabilizes Fe-S cluster proteins, and generates mitochondrial proteotoxic stress [5,60,61].

Protein lipoylation is a mitochondrial post-translational modification required for the activity of several multienzyme complexes, including pyruvate dehydrogenase and alpha-ketoglutarate dehydrogenase. LIAS catalyzes formation of protein-bound lipoic acid, LIPT1 transfers the lipoyl group to specific lysine residues on mitochondrial enzyme subunits, and DLD functions as a shared E3 component within lipoylated dehydrogenase complexes. FDX1 is a central determinant of cuproptosis because it influences mitochondrial copper redox biology and supports the protein lipoylation machinery that generates copper-sensitive substrates [1,2,5,61,62,63,64].

DLAT is particularly relevant because its lipoylated domain provides a direct copper-binding substrate. Copper binding alters DLAT interactions and promotes higher-order complexes, leading to proteotoxic stress and impaired mitochondrial metabolism. In chronic renal fibrosis, mitochondrial copper interacted with lipoylated DLAT, promoted DLAT dimerization, inhibited pyruvate dehydrogenase activity, and contributed to mitochondrial dysfunction, senescence, and fibrosis [9]. This finding links renal mitochondrial copper to a canonical cuproptosis substrate, but the biological outcome may range from sublethal metabolic inhibition to lethal cuproptosis depending on accompanying Fe-S destabilization, pathway dependence, and cell-death rescue [65].

Destabilization of Fe-S cluster proteins is another characteristic component of cuproptosis, but it is not unique to this pathway. In renal ischemia–reperfusion injury, iron overload disrupted Fe-S cluster assembly and impaired LIAS function, increasing tubular sensitivity to copper-associated injury [38]. Because Fe-S proteins are also vulnerable to oxidative stress, iron overload, and generalized mitochondrial damage, their loss should be interpreted together with DLAT aggregation, quantitative protein lipoylation, FDX1 dependence, copper availability, and functional rescue [60,66,67,68,69,70,71,72,73].

**Figure 3:**
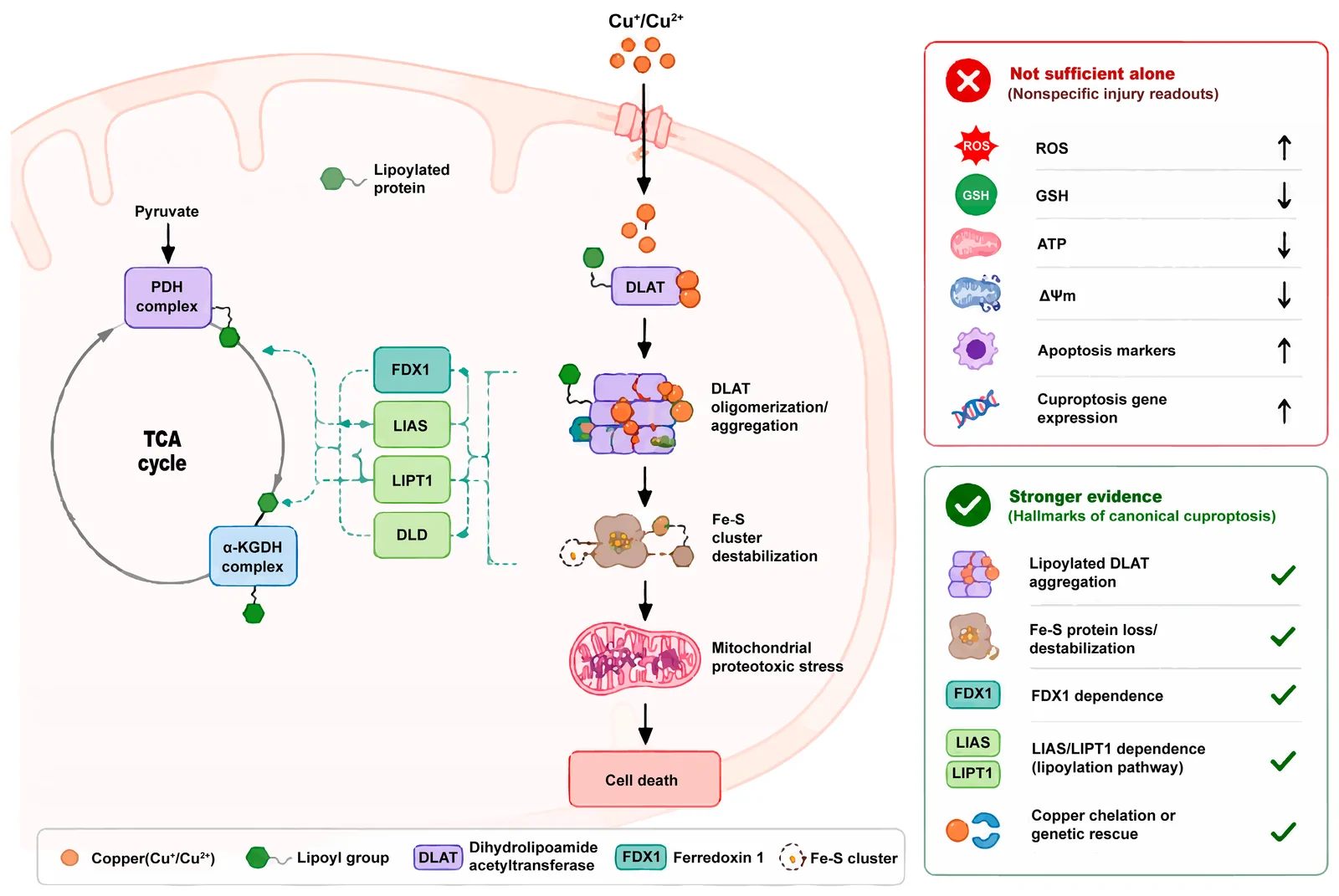
Molecular core and evidentiary criteria of canonical cuproptosis. Canonical cuproptosis is a copper-dependent mitochondrial proteotoxic death pathway initiated by copper binding to lipoylated tricarboxylic acid (TCA)-cycle proteins, particularly DLAT, resulting in DLAT aggregation, Fe-S cluster protein destabilization, and FDX1- and protein lipoylation-dependent mitochondrial stress. Definitive identification of canonical cuproptosis requires evidence of copper-dependent lipoylated protein aggregation, Fe-S protein loss, and genetic or pharmacological dependence on the FDX1–lipoylation axis. In contrast, oxidative stress, glutathione depletion, ATP loss, mitochondrial depolarization, and apoptosis-related markers represent supportive but nonspecific indicators of mitochondrial injury. This distinction is critical for interpreting copper-associated renal injury and avoiding overclassification of general copper toxicity as canonical cuproptosis.

The FDX1-protein lipoylation-DLAT/Fe-S module therefore defines the molecular core of canonical cuproptosis. By contrast, ROS generation, GSH depletion, membrane depolarization, ATP loss, or respiratory-complex inhibition are broader mitochondrial injury processes that may occur upstream, downstream, or independently of cuproptosis [5,15,74]. The molecular core and the evidentiary criteria used throughout this review are summarized in Figure 3.

### 3.3. Cellular and Metabolic Determinants of Susceptibility

Renal cell populations differ substantially in mitochondrial content, respiratory dependence, antioxidant capacity, copper-transport machinery, and protein lipoylation. These features shape whether copper dyshomeostasis produces reversible stress, metabolic reprogramming, senescence, inflammation, or cell death [8,75].

Proximal tubular epithelial cells are especially vulnerable because their transport functions depend heavily on oxidative phosphorylation. Ischemia-reperfusion injury, cisplatin nephrotoxicity, oxalate exposure, and lipid-related injury have each been associated with combinations of SLC31A1-dependent copper accumulation, FDX1/LIAS changes, DLAT oligomerization, mitochondrial dysfunction, and rescue by copper chelation or transporter manipulation [8,37,38,51,76]. The extent to which these findings represent canonical cuproptosis varies among models [77].

Podocytes depend on mitochondrial energy to preserve foot-process architecture and cytoskeletal integrity, whereas endothelial cells must maintain barrier function, angiogenic capacity, and redox homeostasis. Copper-dependent mitochondrial injury has been functionally linked to podocyte damage [40], but direct evidence in renal endothelial cells is limited. These differences underscore the need to identify the affected lineage rather than infer a uniform whole-kidney response [78].

Immune cells and renal cancer cells introduce a different metabolic context. In renal cell carcinoma, glycolytic adaptation, pyruvate dehydrogenase activity, DLAT abundance, and FDX1 regulation influence tumor sensitivity to cuproptosis [19,25]. Conversely, cuproptosis in antitumor CD8+ T cells can impair immune function and reduce the efficacy of immune-checkpoint therapy [18]. The same pathway may therefore be therapeutically desirable in malignant cells but harmful in immune effectors or residual renal parenchyma [79,80,81,82].

Single-cell, single-nucleus, and spatial analyses further show that copper-related molecular signatures are distributed unevenly across nephron segments and disease stages. In DKD, FDX1-, LIAS-, and DLAT-related changes are enriched in metabolically active tubular populations, while other cell-death programs may predominate in different renal cell types [83,84]. Bulk-tissue expression can consequently reflect altered cell composition or metabolic state rather than execution of a uniform death pathway [85].

Overall susceptibility is determined by the intersection of copper trafficking, mitochondrial respiratory activity, protein lipoylation capacity, antioxidant defenses, and cell identity. A copper-dependent lesion that is lethal in one renal population may be adaptive, sublethal, or profibrotic in another [1,2,3,86].

### 3.4. Spectrum of Copper-Related Cellular Outcomes

Copper dyshomeostasis does not inevitably lead to cuproptosis. The outcome depends on copper concentration and duration, chemical form, subcellular localization, metabolic state, and the capacity of the affected cell to buffer or redistribute copper.

Moderate or compartment-restricted abnormalities may primarily alter enzyme activity and signaling. Mitochondrial copper can inhibit complex IV and oxidative phosphorylation without activating the complete cuproptosis pathway [50]; cytosolic copper can impair SOD1 activation and promote oxidative stress and senescence [33]; and Golgi copper can activate LOX and enhance extracellular matrix crosslinking [52]. Copper binding to lipoylated DLAT may likewise inhibit pyruvate dehydrogenase and promote senescence or fibrosis before a lethal threshold is reached [87].

At higher levels or in susceptible metabolic states, copper stress may engage overlapping regulated cell-death programs. ROS accumulation and GSH depletion can favor apoptosis or ferroptosis, while mitochondrial damage and released danger signals can amplify inflammasome activation and inflammatory injury. Ischemia-reperfusion injury, cisplatin nephrotoxicity, DKD, and renal fibrosis often contain mixed apoptotic, ferroptotic, pyroptotic, necroptotic, and cuproptosis-related signals [15,16,35,44,88].

Within this spectrum, canonical cuproptosis is most strongly supported when copper-dependent cell loss is accompanied by aggregation of lipoylated mitochondrial proteins, destabilization of Fe-S proteins, dependence on FDX1 or the LIAS/LIPT1 protein lipoylation machinery, and reversal by genetic or pharmacological manipulation of the pathway [1,2,3,5]. When only copper accumulation, oxidative stress, complex IV inhibition, mitochondrial depolarization, apoptosis, senescence, or gene-expression changes are demonstrated, broader descriptions such as copper dyshomeostasis or copper-associated mitochondrial injury are more accurate [89].

This continuum provides a mechanistic basis for interpreting the disease-specific evidence in the following section. The central questions are not simply whether copper is elevated, but which renal cell is affected, where copper accumulates, which molecular target is altered, whether the lesion is reversible or lethal, and whether the canonical mitochondrial proteotoxic pathway is required [90]. For practical classification, Table 1 contrasts copper-associated injury with canonical cuproptosis and lists the assays most useful for distinguishing them.

## 4. Disease-Specific Evidence for Copper Metabolism-Related Cell Death

### 4.1. Acute Kidney Injury: Ischemia-Reperfusion, Nephrotoxicity and Sepsis

Among nonmalignant renal conditions, acute tubular injury currently provides the most developed mechanistic evidence [1,2,3]. In renal ischemia–reperfusion injury, iron overload intensified tubular injury by disrupting Fe-S cluster assembly and impairing LIAS, whereas iron chelation or restoration of Fe-S assembly attenuated damage [38]. This study links Fe-S biology, LIAS, and metal crosstalk to tubular cell death. Interpretation remains complex, however, because intact protein lipoylation supplies the copper-binding substrates of canonical cuproptosis; simultaneous assessment of residual lipoylation, DLAT aggregation, Fe-S loss, and FDX1 dependence would clarify the temporal sequence [1,2,91,92,93].

In a complementary ischemia–reperfusion model, nitroxyl promoted lysosomal degradation of SLC31A1, reduced copper accumulation and FDX1/LIAS expression, and protected HK-2 cells and mice. Elesclomol reversed this protection, whereas SLC31A1 overexpression prevented the beneficial effect [37]. These genetic and pharmacological reversals support a causal role for copper uptake, although direct analysis of lipoylated-protein aggregation and Fe-S destabilization would strengthen assignment to cuproptosis.

Cisplatin nephrotoxicity has also been linked to copper uptake. ELF3-driven SLC31A1 upregulation increased intracellular Cu+, mitochondrial dysfunction, oxidative stress, and cell death, whereas SLC31A1 knockdown improved cellular and renal outcomes [8]. Thymol and quercetin studies reported improvement in renal injury together with changes in SLC31A1, FDX1, LIAS, DLAT, copper accumulation, oxidative stress, HSP70, or mitochondrial function [94,95]. These studies support copper-dependent tubular injury, but gene expression, HSP70, ROS, or membrane potential alone provide only partial evidence for canonical cuproptosis.

Sepsis-associated AKI and cardiopulmonary bypass-associated AKI extend the evidence toward clinically relevant settings. Reduced GSH lowered renal copper and SLC31A1 and improved sepsis-induced AKI in mice [17]. In diabetic patients undergoing cardiopulmonary bypass, perioperative increases in copper and proposed cuproptosis-related biomarkers were accompanied by animal evidence of mitochondrial injury and altered Fe-S proteins [96]. The human observations remain correlative and require tissue- and cell-resolved validation.

Imaging and computational studies may help identify AKI states in which copper-related injury is active. A near-infrared superoxide probe detected early oxidative changes during ischemia–reperfusion AKI and was used to monitor the effects of cuproptosis-directed intervention [97]. Bioinformatics and machine-learning studies have also identified cuproptosis-related genes in ischemia–reperfusion injury and AKI [98,99,100]. These approaches are useful for biomarker discovery and patient stratification, but expression patterns and redox imaging are not direct evidence of the complete death program.

### 4.2. Crystal-Related and Lipid-Related Tubular Injury

Oxalate-induced crystal injury provides a metabolically relevant tubular model. In glyoxylate-treated mice and oxalate-exposed HK-2 cells, ginsenoside Ro reduced renal injury, macrophage infiltration, oxidative stress, and crystal adhesion. The study assessed DLAT oligomerization, FDX1, HSP70, mitochondrial ROS, membrane potential, and GSH and incorporated ZnT1 gain- and loss-of-function together with tetrathiomolybdate rescue [76]. The combination of DLAT oligomerization and functional rescue provides comparatively strong support, although Fe-S changes and direct FDX1/protein lipoylation dependence remain to be established [101].

Lipid-related kidney injury may also disturb copper export. In biopsy-confirmed kidney disease and high-fat or oxidized-LDL models, higher serum, urinary, and renal copper levels were associated with worse renal function. Oxidized LDL suppressed ATP7B, increased tubular copper accumulation, and promoted mitochondrial dysfunction and proposed cuproptosis-related changes [51]. The findings connect human copper abnormalities with experimental tubular injury, but lipotoxicity, oxidative stress, and apoptosis are important competing mechanisms.

### 4.3. Diabetic Kidney Disease and Podocyte Injury

DKD is a chronic metabolic disease in which copper imbalance, mitochondrial stress, inflammation, and multiple cell-death programs converge. Integrated bulk and single-cell transcriptomics, spatial analysis, urinary copper measurement, and protein validation identified increased FDX1 and LIAS in metabolically active tubular segments of diabetic mice and altered DLAT forms in db/db kidneys [83]. This comprehensive dataset supports a cuproptosis-related molecular state, but in vivo dependence on FDX1/LIAS and rescue of DLAT aggregation and Fe-S loss were not fully established [102,103,104,105,106].

Podocyte studies provide complementary functional evidence. Copper or adriamycin exposure increased intracellular and renal copper, mitochondrial dysfunction, ROS, membrane depolarization, ATP loss, and cytoskeletal injury. Reducing copper accumulation and deleting CXCL5 attenuated the cuproptosis-related phenotype and podocyte damage [40]. Further work is needed to determine whether CXCL5 acts through FDX1 and protein lipoylation and whether lipoylated DLAT aggregation and Fe-S destabilization occur in vivo [107].

Several DKD studies are based primarily on bioinformatics analyses. Cuproptosis-related gene clusters have been associated with immune infiltration, fibrosis, and disease heterogeneity, and machine-learning models have nominated candidate diagnostic genes [22,23,108]. A broader single-nucleus analysis of thirteen cell-death programs identified apoptosis, necroptosis, pyroptosis, and entotic cell death as prominent pathways, indicating that cuproptosis-related signatures may not dominate every dataset [84]. These studies are valuable for hypothesis generation and cell-type prioritization, but not for establishing execution of cuproptosis [109].

### 4.4. Chronic Kidney Disease, Renal Fibrosis and End-Stage Renal Disease

Evidence in chronic CKD and renal fibrosis is biologically important but mechanistically incomplete. Clinical and genetic analyses have associated higher serum copper with CKD risk, lower estimated glomerular filtration rate, and more severe biopsy-defined fibrosis [58,110,111]. However, the observational literature is heterogeneous: copper intake was inversely associated with CKD and DKD within a limited intake range, a systematic review found no significant serum-copper difference in CKD overall and lower concentrations in hemodialysis, and individual hemodialysis cohorts reported variable copper status [112,113,114]. These findings support an exposure-disease relationship but do not identify the responsible cellular injury pathway [4].

The most cuproptosis-adjacent fibrosis study demonstrated increased mitochondrial copper in fibrotic kidneys, direct interaction with lipoylated DLAT, altered DLAT subunit assembly, DLAT dimerization, inhibition of pyruvate dehydrogenase, and subsequent mitochondrial dysfunction, senescence, and fibrosis. Lowering mitochondrial copper restored pyruvate dehydrogenase activity and attenuated fibrosis [9]. This work directly links mitochondrial copper to a canonical cuproptosis substrate. Nevertheless, the dominant outcomes were enzyme inhibition, senescence, and fibrosis, while Fe-S destabilization, FDX1/LIAS/LIPT1 dependence, and death-specific rescue were not fully established. The findings are therefore best interpreted as a copper-DLAT mechanism that may overlap with, but does not yet prove, lethal cuproptosis during CKD progression [115].

Other fibrosis studies define parallel copper-dependent mechanisms. Mitochondrial copper accumulation inhibited respiratory complex IV, whereas COX17 preserved copper homeostasis and protected against mitochondrial dysfunction, apoptosis, and fibrosis [50]. Copper overload also impaired SOD1 homodimerization through COMMD1, promoting oxidative stress, senescence, and fibrosis [33]. In the Golgi, ATP7A and FBLN4 delivered copper to LOX and enhanced extracellular matrix crosslinking [52]. Earlier work similarly linked CTR1-dependent copper influx to LOX activation and showed that copper chelation reduced fibrosis [116,117]. These pathways are central to copper-dependent fibrosis but are mechanistically distinct from canonical cuproptosis [118,119,120,121,122,123,124].

Bioinformatics studies provide an additional, lower-resolution evidence layer. UUO datasets have shown altered cuproptosis scores and immune pathways [125], and peripheral-blood transcriptomics in end-stage renal disease has identified differential expression of cuproptosis-related genes and immune-associated clusters [126]. These signatures may nominate biomarkers or molecular subtypes, but they can also reflect systemic inflammation, altered leukocyte composition, dialysis-related factors, or uremia rather than death of renal parenchymal cells [127].

Taken together, copper dyshomeostasis contributes to CKD and renal fibrosis through mitochondrial, redox, senescence, and extracellular matrix pathways, and direct interaction between copper and lipoylated DLAT has been demonstrated [9,33,50,52]. Canonical cuproptosis, however, has not yet been comprehensively established as a driver of renal fibrosis or CKD progression. Resolving this question is a central research priority rather than a settled conclusion [125,126,128].

### 4.5. Renal Malignancies and Tumor Immunity

Renal malignancies provide a distinct experimental setting because therapeutic studies deliberately manipulate copper availability and mitochondrial metabolism. In clear cell renal cell carcinoma (ccRCC), USP15 stabilized c-Myc, increased PDK1/3/4, suppressed DLAT and pyruvate dehydrogenase activity, and conferred resistance to cuproptosis and sunitinib. USP15 depletion or copper-ionophore strategies restored sunitinib sensitivity in vitro and in vivo [25], linking metabolic state, DLAT regulation, drug resistance, and functional cuproptosis manipulation [129,130,131,132,133,134].

Additional studies have targeted tumor cells or the tumor microenvironment. PIK-III-mediated thiamine elevation resensitized renal cell carcinoma to cuproptosis through PDHA1 activation [135]. A pH-responsive hydrogel delivering sunitinib and copper oxide nanoparticles induced mitochondrial proteotoxicity and Fe-S protein depletion while remodeling the immune microenvironment [19]. These findings support therapeutic induction of cuproptosis in renal tumors, but they cannot be directly extrapolated to nonmalignant renal parenchyma, where preservation of viable cells is the primary objective [136,137,138,139].

The immune compartment adds a further layer of complexity. Iron-associated FDX1 stabilization can promote cuproptosis in CD8+ T cells and reduce immunotherapy efficacy, whereas tumor-directed copper delivery may enhance immunogenic tumor-cell death and immune reprogramming [18,19]. Effective renal cancer therapy will therefore require cell-selective approaches that enhance cuproptosis in malignant cells while limiting injury to effector lymphocytes, vascular cells, and residual renal tissue [140,141].

### 4.6. Inherited and Systemic Copper Disorders with Renal Involvement

**Figure 4:**
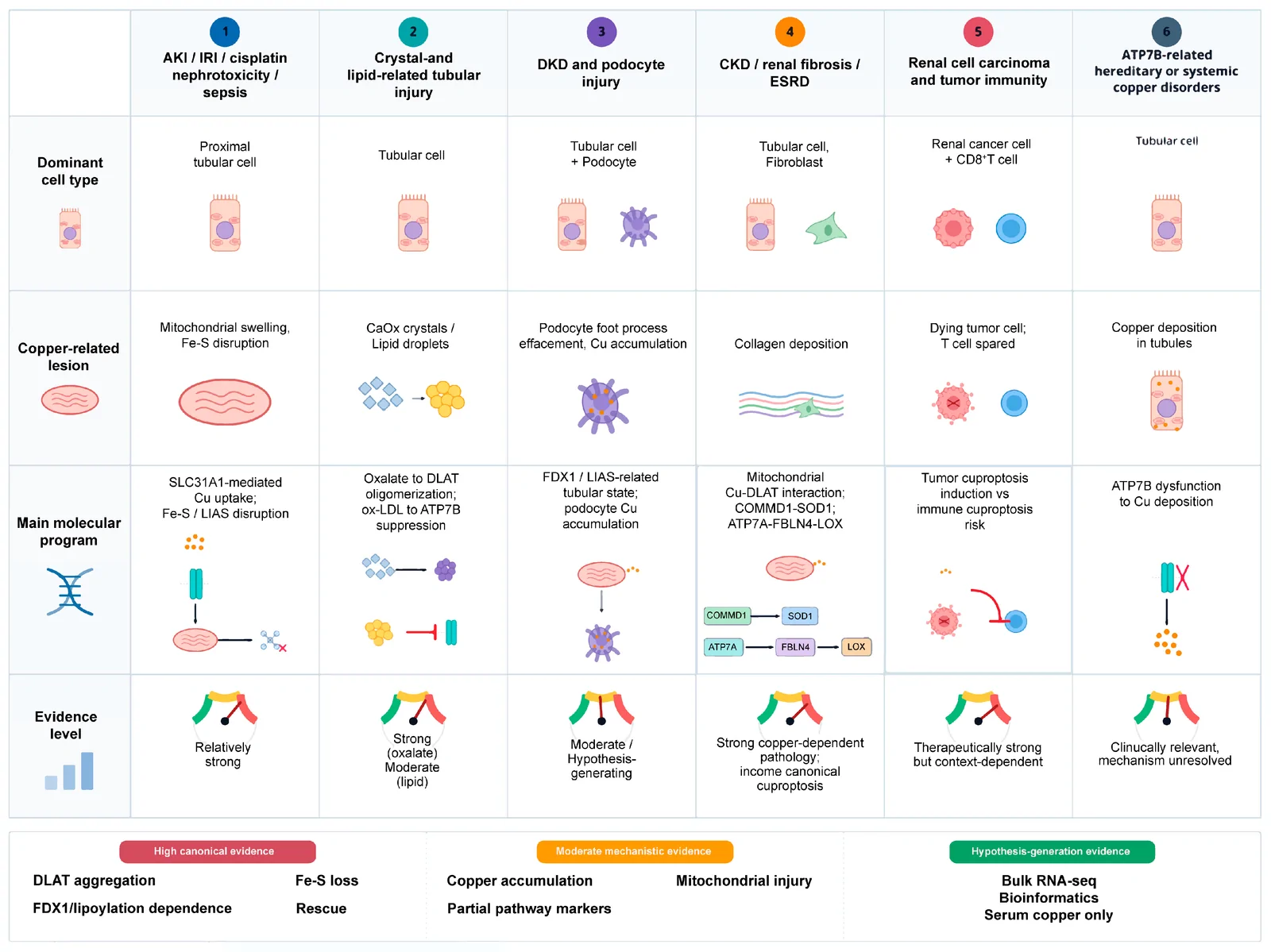
Disease- and cell-type-specific copper-related injury programs across kidney diseases. Copper-related renal injury varies according to disease context, cell lineage, metabolic state, and subcellular copper distribution. Acute tubular injury, crystal-induced tubular injury, and renal cell carcinoma models currently provide relatively stronger evidence for activation or therapeutic exploitation of cuproptosis-related machinery, whereas diabetic kidney disease, CKD/renal fibrosis, and ATP7B-related copper disorders show copper accumulation and copper-dependent mitochondrial, redox, senescence, and matrix-remodeling pathways that are mechanistically important but not yet sufficient to establish fully canonical cuproptosis. Evidence strength is graded according to the presence of DLAT aggregation, Fe-S protein loss, FDX1/lipoylation dependence, and rescue by copper chelation or pathway modulation.

Wilson disease and ATP7B-related disorders illustrate how abnormal copper handling can directly affect the kidney. A renal-limited case involving a heterozygous ATP7B variant showed marked copper deposition in tubular epithelial cells and salt-wasting CKD without a classic systemic Wilson disease phenotype [56]. Broader models emphasize copper speciation, interorgan crosstalk, barrier dysfunction, and tubular injury [57]. Histological copper deposition is clinically relevant, but it does not distinguish cuproptosis from oxidative injury, apoptosis, or adaptive tubular dysfunction [142]. The disease- and cell-type-specific evidence is integrated visually in Figure 4 and compared in detail in Table 2.

## 5. Mechanistic Integration Across Kidney Diseases

### 5.1. Shared Redox-Mitochondrial Injury Programs

Across kidney diseases, redox imbalance and mitochondrial dysfunction form a recurrent injury program, but the initiating lesion and downstream outcome vary with disease context. In acute tubular injury, ischemia, cisplatin exposure, and increased SLC31A1-mediated copper uptake can rapidly increase mitochondrial stress, ROS production, membrane depolarization, and ATP loss [8,37,38]. In chronic fibrosis, sustained mitochondrial copper accumulation has been linked instead to complex IV inhibition, copper-DLAT-mediated suppression of pyruvate dehydrogenase, COMMD1-SOD1 dysfunction, senescence, and progressive matrix remodeling [9,33,50]. Acute cell loss and chronic maladaptive remodeling therefore share biological components but are not equivalent outcomes [143].

Crystal- and lipid-related tubular injury illustrate the same principle. Oxalate-associated injury combines mitochondrial ROS, GSH depletion, DLAT oligomerization, inflammation, and tubular damage, whereas oxidized-LDL-associated injury combines ATP7B suppression, copper accumulation, lipotoxicity, and mitochondrial dysfunction [51,76]. In renal fibrosis, ATP7A-dependent Golgi delivery of copper to LOX translates intracellular copper dysregulation into extracellular matrix crosslinking without requiring mitochondrial cuproptotic death [52]. Similar readouts can therefore arise from distinct copper-dependent lesions in different renal compartments [144].

ROS accumulation, GSH depletion, ATP loss, and mitochondrial structural abnormalities should consequently be interpreted as contextual evidence rather than stand-alone markers of cuproptosis. These changes can accompany apoptosis, ferroptosis, inflammatory signaling, and generalized mitochondrial failure, and complex IV inhibition can occur independently of the FDX1-protein lipoylation-DLAT pathway [5,15,35,50]. Their mechanistic significance depends on the presence or absence of lipoylated-protein aggregation, Fe-S destabilization, pathway dependence, and rescue [145].

### 5.2. Context-Dependent Engagement of the Cuproptosis Machinery

Metabolically active tubular epithelium currently provides the most developed nonmalignant evidence for engagement of the cuproptosis machinery. Ischemia-reperfusion studies have implicated Fe-S/LIAS disruption and SLC31A1-dependent copper accumulation, whereas oxalate injury has demonstrated DLAT oligomerization and rescue by copper chelation [37,38,76]. Cisplatin studies further support an ELF3-SLC31A1 copper-uptake axis, although mitochondrial dysfunction, apoptosis, and oxidative stress remain prominent coexisting processes [8,95].

Chronic metabolic and glomerular diseases show a more heterogeneous pattern. In DKD, integrated multi-omics analyses localize FDX1/LIAS- and DLAT-related changes to metabolically active nephron segments, but direct in vivo pathway dependence and rescue remain incomplete [83]. In podocytes, copper reduction and CXCL5 deletion preserve mitochondrial function and cytoskeletal integrity, supporting a functional copper-dependent injury program whose relationship to FDX1 and protein lipoylation requires further definition [83]. In fibrosis, copper binding to lipoylated DLAT provides a mechanistic point of contact with cuproptosis, while complex IV inhibition, COMMD1-SOD1 dysfunction, and ATP7A-FBLN4-LOX activation represent parallel copper-dependent pathways [9,33,50,52,146].

Renal malignancy demonstrates how metabolic state can redirect the same molecular machinery toward a different therapeutic goal. Glycolytic adaptation and suppression of DLAT/pyruvate dehydrogenase activity can confer resistance to cuproptosis in ccRCC, whereas restoration of mitochondrial metabolic susceptibility or targeted copper delivery can resensitize tumor cells [19,25]. At the same time, FDX1 stabilization can drive cuproptosis in CD8+ T cells and impair antitumor immunity [18].

Thus, engagement of the cuproptosis machinery is shaped by cell identity, mitochondrial respiratory dependence, protein lipoylation capacity, antioxidant status, and the subcellular destination of copper. This framework explains why acute tubular injury, podocyte damage, chronic fibrosis, and renal cancer do not display a uniform molecular pattern and why bulk-tissue or transcriptomic signals cannot substitute for cell-resolved functional evidence [1,2,3,84,147].

### 5.3. Crosstalk Between Cuproptosis and Other Regulated Cell-Death Programs

Cuproptosis occurs within a renal injury environment in which apoptosis, ferroptosis, pyroptosis, and necroptosis may be activated simultaneously or sequentially. Ferroptosis shares GSH depletion, metal-dependent redox stress, and mitochondrial metabolic disturbance, but is defined by iron-dependent phospholipid peroxidation and GPX4 failure. Pyroptosis is mediated by inflammasome-caspase-gasdermin signaling, necroptosis depends on RIPK1/RIPK3/MLKL, and apoptosis involves caspase activation and mitochondrial outer-membrane permeabilization [16,35,88]. Single-nucleus studies in DKD further indicate that the relative contribution of these programs varies among renal cell types and disease stages [84,148,149,150].

This overlap complicates interpretation of pharmacological rescue. Copper chelators can simultaneously reduce labile copper, ROS, mitochondrial injury, apoptosis, and ferroptotic stress, whereas antioxidants or GSH supplementation may improve renal function without interrupting lipoylated-protein aggregation [5,8,17]. Improvement after a chelator, antioxidant, or mitochondrial protective agent should therefore be assigned to cuproptosis only when the characteristic biochemical changes and pathway dependencies are also reversed.

Temporal, cell-resolved, and orthogonal perturbation studies offer a more informative approach. Copper exposure and lipoylated-protein aggregation should be interpreted alongside Fe-S proteins, cell viability, and markers of apoptosis, ferroptosis, pyroptosis, and necroptosis. Genetic perturbation can then establish whether cuproptosis is necessary, sufficient, parallel, or secondary within the overall injury response. Transcriptomic, single-cell, and machine-learning analyses can prioritize candidate cells and pathways, but causal assignment requires functional validation [16,98,99,151].

This network view also informs therapy. In nonmalignant kidney disease, correcting an upstream defect in copper uptake, export, or compartmentalization may suppress several downstream injury programs. Direct modulation of FDX1 or protein lipoylation is more appropriate when the canonical pathway has been demonstrated. In renal cancer, combination strategies may exploit tumor-cell cuproptosis while protecting immune and parenchymal cells [152].

## 6. Therapeutic and Translational Implications

### 6.1. Suppressing Copper-Dependent Injury in Nonmalignant Kidney Disease

Potential strategies for nonmalignant kidney disease include copper chelation, reduction in SLC31A1-mediated uptake, enhancement of ATP7A/ATP7B-dependent export, correction of organelle-specific copper trafficking, restoration of GSH and antioxidant capacity, preservation of Fe-S cluster biology, and support of mitochondrial quality control. Tetrathiomolybdate, D-penicillamine, and other copper-modulating agents have shown preclinical renal effects, while transporter-directed interventions have produced genetic rescue in several models [8,37,99,117,153].

Therapeutic selection should follow the demonstrated lesion. In ATP7A-FBLN4-LOX-driven fibrosis, inhibition of LOX activation or secretory-pathway copper transfer may be more rational than broad suppression of FDX1 [52]. When COMMD1-mediated SOD1 dysfunction predominates, restoring SOD1 activity or modulating COMMD1 may be preferable [33]. Direct modulation of FDX1, protein lipoylation, or mitochondrial copper is most relevant when canonical cuproptosis has been established [1,5,154].

### 6.2. Exploiting Cuproptosis in Renal Cancer

In renal cancer, the therapeutic direction is reversed. Copper ionophores, restoration of mitochondrial metabolism, suppression of glycolytic escape, reactivation of DLAT/pyruvate dehydrogenase, and targeted copper delivery can increase tumor susceptibility to cuproptosis. Studies of sunitinib resistance and copper-delivery platforms support this strategy [19,25]. Cell-selective delivery remains essential because systemic manipulation of copper may also affect renal parenchymal, vascular, and immune cells [1,18,155,156,157,158,159,160].

### 6.3. Biomarkers, Safety and Translational Constraints

Candidate biomarkers reported in kidney and copper-metabolism studies include circulating or urinary copper, copper-to-zinc ratios, SLC31A1/ATP7A/ATP7B expression, FDX1 and proteins involved in mitochondrial lipoylation, lipoylated DLAT aggregates, Fe-S protein changes, exploratory proteotoxic-stress markers, and imaging-based redox signals [76,83,96,97,110,161]. No single measurement is diagnostic. A translational panel should combine copper exposure, a substrate- or proteostasis-based readout, cell injury, and renal function, ideally with spatial localization in human tissue [112,113,162].

**Figure 5:**
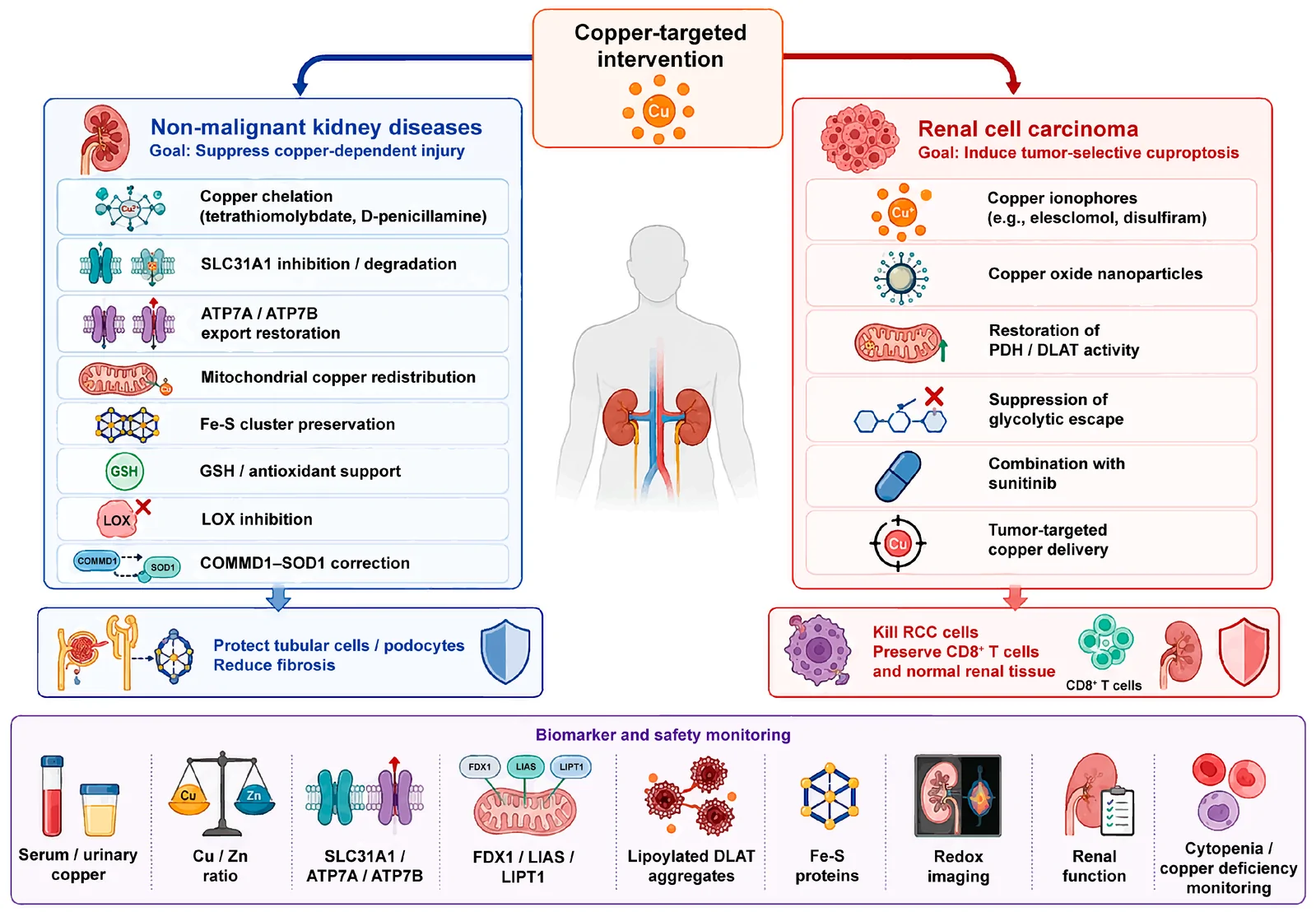
Translational roadmap for copper-targeted diagnosis and therapy in kidney diseases. Therapeutic strategies should be matched to the demonstrated copper-dependent lesion rather than applied as nonspecific copper reduction. Nonmalignant kidney diseases may benefit from suppressing copper uptake, correcting copper export or organelle trafficking, preserving antioxidant and Fe-S cluster biology, or inhibiting copper-dependent matrix remodeling. In renal cell carcinoma, tumor-selective copper delivery or cuproptosis induction may enhance anticancer efficacy. Across both settings, biomarker-guided patient stratification and strict safety monitoring are required to avoid systemic copper depletion, cytopenia, immune-cell injury, vascular toxicity, and damage to residual renal parenchyma.

Safety is a major translational constraint because both copper excess and copper deficiency are harmful. Copper is required for mitochondrial respiration, antioxidant defense, extracellular matrix maturation, hematopoiesis, and immune function [1,57]. Chronic copper depletion can cause clinically important cytopenias, and CKD has been identified as a risk factor for zinc-induced copper deficiency [163]. Conversely, a high copper-to-zinc ratio is associated with oxidative stress and anemia in cardiovascular-kidney-metabolic disease [161]. Dose, timing, disease stage, baseline copper status, renal clearance, and cellular targeting must therefore be incorporated into therapeutic development [164,165,166,167]. These diagnostic, therapeutic, and safety considerations are integrated into the translational roadmap in Figure 5.

## 7. Conclusions and Future Perspectives

Copper dyshomeostasis is an increasingly well-supported component of kidney disease biology. Through effects on mitochondrial respiration, redox balance, protein lipoylation, antioxidant enzymes, and extracellular matrix maturation, abnormal copper handling can contribute to tubular and podocyte injury, inflammation, senescence, fibrosis, and tumor metabolic vulnerability across AKI, CKD, DKD, nephrolithiasis, hereditary copper disorders, and renal cancer [1,2,3,15]. The broader concept of copper metabolism-related cell death is useful because it accommodates these diverse outcomes without assuming a single death mechanism [168].

Canonical cuproptosis is a narrower entity defined by copper-dependent aggregation of lipoylated mitochondrial proteins, destabilization of Fe-S proteins, and dependence on FDX1 and the protein lipoylation pathway [5]. The strongest renal evidence currently comes from selected acute tubular, crystal-injury, and renal cancer models [25,37,38,76]. In chronic CKD and fibrosis, copper-DLAT interaction, complex IV inhibition, COMMD1-SOD1 dysfunction, and ATP7A-FBLN4-LOX activation establish important copper-dependent pathology, but do not yet demonstrate a complete FDX1/protein lipoylation-dependent lethal pathway [9,33,50,52]. These findings support a major role for copper metabolism but do not yet comprehensively demonstrate canonical FDX1/lipoylation-dependent cuproptotic death during CKD progression.

The central unresolved question is whether canonical cuproptosis contributes causally to chronic CKD progression or whether most chronic lesions reflect parallel copper-dependent processes such as respiratory inhibition, redox failure, senescence, and matrix crosslinking. Addressing this question will require complementary chronic models, longitudinal sampling from early injury to established fibrosis, and cell-specific manipulation of Fdx1, Lias, Lipt1, Dlat, Slc31a1, Atp7a, or Atp7b [9,40,83,125].

Mechanistic studies should integrate organelle-resolved copper measurements with quantitative protein lipoylation, lipoylated DLAT aggregation, Fe-S protein abundance or activity, mitochondrial respiration, and direct assessment of cell viability. Pharmacological chelation is most informative when paired with genetic perturbation, because chelators also suppress nonspecific oxidative injury. Conversely, copper supplementation or ionophore treatment should be accompanied by evidence of target engagement and compartment-specific copper accumulation [1,5,37,38].

A second priority is to resolve mixed cell-death phenotypes. Ischemia-reperfusion injury and DKD involve overlapping apoptosis, ferroptosis, pyroptosis, necroptosis, and other death programs [16,35,84]. Combining pathway-specific inhibitors or genetic models with temporal and single-cell analyses will help determine whether cuproptosis initiates injury, amplifies another pathway, or accounts for only a fraction of total cell loss. Multi-omics can identify candidate pathways, but functional epistasis remains necessary for causal inference [98,99,169].

Human validation must extend beyond serum copper and transcriptomic signatures. Current evidence includes associations between copper exposure and fibrosis, copper-to-zinc imbalance and anemia, peripheral-blood cuproptosis-related signatures in end-stage renal disease, and renal copper deposition associated with ATP7B dysfunction [56,110,126,161]. Prospective studies should relate copper species and renal function to tissue localization, lipoylated DLAT aggregation, Fe-S destabilization, and cell-type-specific FDX1/protein lipoylation activity. Archived renal biopsies and paired blood or urine samples may provide practical initial platforms for this work.

Finally, consistent terminology and reporting will be essential for comparing studies. “Copper dyshomeostasis” or “copper-associated mitochondrial injury” should be used when evidence is limited to copper levels, ROS, GSH, complex IV, apoptosis, or gene-expression signatures. “Cuproptosis” should be reserved for studies that demonstrate multiple canonical features together with causal rescue [1,2,5]. This convention preserves mechanistic precision without excluding biologically important copper-dependent processes that fall outside canonical cuproptosis. Accordingly, Table 3 outlines a practical experimental framework for evaluating whether canonical cuproptosis contributes to chronic CKD and renal fibrosis.

Progress will depend on cell- and organelle-resolved measurements, integrated biochemical and genetic validation, and explicit separation of cuproptosis from other regulated cell-death programs. Such studies should clarify when copper should be chelated, redistributed, buffered, or deliberately delivered and may support mechanism-based biomarkers and therapies tailored to different kidney diseases.

## Figures and Tables

**Table 1:** Distinguishing copper-associated injury from canonical cuproptosis.

Feature	Copper-Associated Injury	Canonical Cuproptosis	Interpretive Value	Recommended Assay
Copper burden	Elevated total or labile copper in tissue or cells	Required trigger, but not sufficient	Supportive	ICP-MS, validated copper probes, or subcellular fractionation
Oxidative stress	Increased ROS, GSH depletion, and lipid or protein oxidation	May occur upstream or downstream	Nonspecific	mtROS, GSH/GSSG, MDA/4-HNE, or redox-proteomic readouts
Mitochondrial dysfunction	Membrane depolarization, ATP loss, or respiratory-complex inhibition	Common consequence	Nonspecific	Respiratory flux, membrane potential, ATP, and complex activity
Lipoylated-protein aggregation	Usually absent or untested	Characteristic biochemical feature	High specificity	Nonreducing immunoblot and complementary DLAT aggregation assays
Fe-S protein destabilization	May occur during generalized oxidative injury	Characteristic pathway component	High specificity when combined with other criteria	Representative Fe-S proteins and enzyme activities
FDX1/protein lipoylation dependence	Not required	Expected mechanistic dependence	Causal criterion	FDX1, LIAS, or LIPT1 perturbation and rescue
Rescue	Chelators or antioxidants may improve injury nonspecifically	Copper-directed and pathway-specific rescue reverses both hallmarks and cell death	Essential for causality	Copper chelation, pathway rescue, and ionophore reversal
Competing death pathways	Apoptosis, ferroptosis, pyroptosis, or necroptosis may coexist	Relative contributions should be experimentally resolved	Essential for classification	Caspase, GPX4/lipid ROS, GSDMD, and RIPK3/MLKL readouts with pathway-specific perturbation

**Table 2:** Disease-specific evidence for cuproptosis and other copper-dependent mechanisms in kidney disease.

Disease/Model	Main Cell/Context	Key Evidence	Evidence Tier	Main Limitation
Renal ischemia–reperfusion injury	Tubular epithelium	Fe-S/LIAS perturbation; SLC31A1 manipulation; ionophore reversal	Tier 1–2: mechanistic, but incomplete	Simultaneous validation of DLAT aggregation, Fe-S destabilization, and FDX1 dependence is lacking
Cisplatin AKI	Tubular epithelium	Cu+ accumulation; ELF3-SLC31A1 axis; knockdown rescue; proposed CRG changes	Tier 2: partial mechanistic evidence	Mitochondrial dysfunction and apoptosis may coexist; canonical hallmarks incomplete
Sepsis/CPB AKI	Whole kidney; tubular injury	Copper/SLC31A1 changes; GSH or clinical biomarker associations	Tier 2–3	Mostly redox or association endpoints
Kidney stone/crystal injury	HK-2/tubular epithelium	DLAT oligomerization; ZnT1 manipulation; TTM rescue	Tier 1–2	Fe-S and direct FDX1/lipoylation dependency require confirmation
Lipid-related renal injury	Tubular epithelium	Human copper association; ATP7B suppression; mitochondrial injury	Tier 2–3	Lipotoxicity and oxidative death are major confounders
DKD	Tubular segments; glomerulus	Multi-omics localization; urinary copper; FDX1/LIAS and DLAT changes	Tier 2: partial evidence	No full functional rescue or Fe-S validation
Podocyte injury/CKD	Podocytes	Copper reduction and CXCL5 deletion rescue cytoskeletal injury	Tier 2: partial evidence	Canonical substrate and Fe-S hallmarks incomplete
Renal fibrosis/CKD	Tubules, fibroblasts, whole kidney	Copper-DLAT dimerization/PDH inhibition; complex IV; COMMD1-SOD1; ATP7A-FBLN4-LOX	Tier 2 for cuproptosis-adjacent findings; Tier 3 for other copper mechanisms	Full lethal cuproptosis not established
ESRD/UUO signatures	Peripheral blood or bulk kidney	CRG clusters, machine learning, cuproptosis scores	Tier 4: association only	Expression signatures are not cell-death evidence
ccRCC	Tumor cells and immune cells	DLAT/PDH regulation; ionophore sensitivity; in vivo rescue; Fe-S/proteotoxic therapy	Tier 1–2 in selected tumor models	Tumor context and cell-type-specific effects limit extrapolation
ATP7B-related renal copper deposition	Tubular epithelium	Biopsy copper deposition and transporter defect	Tier 3: copper-associated injury	No canonical cuproptosis analysis

**Table 3:** Experimental framework for evaluating cuproptosis in chronic CKD and renal fibrosis.

Domain	Minimum Requirement	Preferred Approach	Interpretive Purpose
Copper exposure	Total and labile copper	Organelle fractionation with quantitative copper measurement or validated imaging	Establish the trigger and relevant compartment
Canonical substrate	Abundance and aggregation of lipoylated DLAT	Nonreducing immunoblot, complementary aggregation assays, imaging, or proteomic analysis	Demonstrate substrate-specific proteotoxicity
Fe-S biology	Changes in multiple Fe-S proteins or enzyme activities	Representative Fe-S proteins combined with functional enzyme assays	Establish characteristic Fe-S destabilization
Genetic dependence	FDX1 or LIAS/LIPT1 perturbation	Cell-specific knockdown, knockout, or rescue	Demonstrate pathway necessity
Pharmacological rescue	Copper chelation and copper/ionophore reversal	Dose–response analysis with evidence of target engagement	Link copper exposure to the phenotype
Cell death	Direct quantification of viability or cell loss	Live-cell analysis, histology, renal function, and lineage-resolved assessment where feasible	Distinguish lethal injury from sublethal senescence or dysfunction
Competing pathways	At least apoptosis and ferroptosis	Caspase, GPX4/lipid ROS, GSDMD, and p-MLKL readouts with appropriate inhibitors or genetic models	Quantify pathway specificity and crosstalk
Human relevance	Validation in patient tissue or biofluids	Spatially resolved tissue assays with paired blood or urine measurements	Support translation beyond experimental models

## Data Availability

No new data were created or analyzed in this study. Data sharing is not applicable to this article.
